# Triglyceride-glucose index is associated with gastroesophageal reflux disease and erosive reflux disease: a health checkup cohort study

**DOI:** 10.1038/s41598-022-25536-0

**Published:** 2022-12-05

**Authors:** Young Min Kim, Yuna Kim, Jie-Hyun Kim, Jong Suk Park, Su Jung Baik, Jaeyoung Chun, Young Hoon Youn, Hyojin Park

**Affiliations:** 1 Division of Gastroenterology, Department of Internal Medicine, Gangnam Severance Hospital, Yonsei University College of Medicine, 211 Eonjuro, Gangnam-gu, Seoul, 06273 Korea; 2 Division of Endocrinology, Department of Internal Medicine, Gangnam Severance Hospital, Yonsei University College of Medicine, Seoul, Korea; 3 Department of Healthcare Research Team, Health Promotion Center, Gangnam Severance Hospital, Yonsei University College of Medicine, Seoul, Korea

**Keywords:** Gastro-oesophageal reflux disease, Endocrine system and metabolic diseases

## Abstract

The triglyceride-glucose (TyG) index was proposed as a useful marker of metabolic syndrome. Insulin resistance, the main mechanism underlying metabolic syndrome, is related to gastroesophageal reflux disease (GERD). This study aimed to elucidate the association between the TyG index and GERD/erosive reflux disease (ERD). We retrospectively reviewed the electronic medical records of patients who underwent gastroduodenoscopy at a checkup center. The calculation of TyG index used following formula: ln (fasting triglycerides [mg/dL] × fasting glucose [mg/dL]/2). We divided the patients into four groups according to the TyG index quartile (Q). We evaluated the relationship between the alteration of the TyG index and GERD in patients who received health checkups two times. Among the 52,605 enrolled patients, 3073 (5.8%) and 434 (0.8%) were diagnosed with GERD and ERD, respectively. The odds ratios (ORs) for GERD in the TyG index progressively increased across quartiles (*P* < 0.001): Q2 (OR = 2.477), Q3 (OR = 3.013), and Q4 (OR = 4.027) compared with Q1, which was used as a reference, respectively. Those for ERD also progressively increased across quartiles (*P* < 0.001): Q2 (OR = 4.264), Q3 (OR = 4.841), and Q4 (OR = 7.390) compared with Q1, respectively. Moreover, the degree of TyG index increase during the first and second tests in the GERD group was more prominent than in the control group (*P* = 0.001). In conclusion, the higher TyG index was significantly associated with GERD. The TyG index may be a novel predictive biomarker of GERD and ERD.

## Introduction

Gastroesophageal reflux disease (GERD) is a common upper gastrointestinal disorder and can be differentiated an erosive esophagitis (esophageal reflux disease, ERD) and non-erosive reflux disease (NERD). The prevalence of GERD in western countries is higher at approximately 20% than that in Asian countries^1,2^. Because of Westernized diets and increased *Helicobacter pylori* (*H. pylori*) eradication rates, the prevalence of GERD in Asian countries has increased by approximately 15%^3–5^. Therefore, GERD is a health concern worldwide. GERD affects patients’ quality of life because of its various clinical symptoms^6^ and GERD-related complications such as Barrett’s esophagus and adenocarcinoma of the esophagus^7^. Therefore, evaluating the predictive factors and understanding the precise pathophysiology of GERD is important.

A previous study reported that the severity of reflux esophagitis is associated with components of metabolic syndrome such as obesity, hyperglycemia, and elevated BP and triglyceride (TG) levels^8^. In addition to these factors, sarcopenia, defined as skeletal muscle attenuation and associated with metabolic syndrome, was an independent risk factor for GERD, including ERD, in a recent study^9^. Interleukin-6 (IL-6) stimulates the secretion of hepatic TGs and plays a role in insulin resistance (IR) at the cellular level in hepatocytes^10,11^. Moreover, the expression of IL-6 was consistently increased in Barrett’s esophagus compared to that in control tissues^12^. Thus, GERD is associated with metabolic syndrome.

In 2008, the triglyceride-glucose (TyG) index was introduced as a novel surrogate marker for IR in healthy individuals for the first time^13^. Previous studies have mainly evaluated the association between the TyG index and diabetes or cardiovascular diseases^14,15^. The TyG index was calculated using a simple formula based on the TG and glucose levels in a routine laboratory test. The TyG index has clinical significance in various metabolic conditions because of the synergistic effect of lipotoxicity and glucotoxicity. Increased adipose tissue causes lipid overflow and inflammation via alteration of the secretion of adipokines and cytokines, thereby playing an essential role in the development of IR^16^. Both lipotoxicity and glucotoxicity have vicious cycles that contribute to reducing the action of insulin on glucose metabolism^17^.

Since the TyG index and GERD are related to metabolic syndrome, we hypothesized that the TyG index is associated with GERD and ERD. However, studies conducted to identify this relationship are scarce. This study aimed to elucidate the predictive factors for GERD, focusing on the TyG index in a large health checkup cohort. In addition, we analyzed the ERD group, which represents a more specific mucosal injury for GERD.

## Results

### Baseline characteristics

Table 1 shows the baseline characteristics of the study population. The median age of the enrolled patients was 50.0 years, and 52.9% of the patients were men. The median BMI was 23.6 kg/m^2^, and 31.8% were diagnosed with obesity. The median TyG index was 9.3. Approximately 11.0% of the patients had hypertension. Additionally, 27.1% of the patients had *H. pylori* infection. A total of 3073 (5.8%) and 434 (0.8%) patients were diagnosed with GERD and ERD, respectively. Other baseline characteristics of the study population are presented in Table 1.

**Table 1:** Baseline characteristics of the study population.

Characteristics	All patients (N = 52,605)
Age, median (IQR), years	50.0 (42.0–57.0)
Male (n, %)	27,802 (52.9)
Height, median (IQR), cm	166.3 (159.7–172.8)
Weight, median (IQR), kg	65.4 (56.1–74.5)
BMI, median (IQR), kg/m^2^	23.6 (21.5–25.8)
Waist, median (IQR), cm	82.0 (74.0–89.0)
SBP, median (IQR), mmHg	120.0 (110.0–13.0)
DBP, median (IQR), mmHg	71.0 (65.0–79.0)
Obesity (n, %)	16,721 (31.8)
TyG index, median (IQR)	9.3 (8.9–9.7)
Current smoker (n, %)	18,882 (35.9)
Alcohol history (n, %)	31,026 (59.0)
Hypertension (n, %)	5767 (11.0)
*H. pylori* infection (n, %)	14,264 (27.1)
**Endoscopic findings**	
LA-A (n, %)	2639 (5.0)
LA-B (n, %)	410 (0.8)
LA-C (n, %)	24 (0.05)
LA-D (n, %)	0 (0.0)
GERD (n, %)	3073 (5.8)
ERD (n, %)	434 (0.8)
**Laboratory finding, median (IQR)**	
Fasting glucose, mg/dL	97.0 (91.0–105.0)
Total cholesterol, mg/dL	201.0 (177.0–227.0)
Triglyceride, mg/dL	109.0 (79.0–159.0)
HDL cholesterol, mg/dL	54.0 (46.0–64.0)
LDL cholesterol, mg/dL	130.0 (109.0–153.0)

*IQR* interquartile range; *BMI* body mass index; *SBP* systolic blood pressure; *DBP* diastolic blood pressure; *TyG index* triglyceride-glucose index; *H. pylori Helicobacter pylori*; *LA* Los Angeles; *GERD* gastroesophageal reflux disease; *ERD* definite erosive reflux disease; *HDL* high-density lipoprotein; *LDL* low-density lipoprotein.

### Risk factors for GERD

In the univariate analysis, males, obesity, smoking, alcohol consumption, and hypertension were significantly associated with GERD (Table 2). In addition, the proportion of *H. pylori* infection in the GERD group was significantly lower than in the control group. The median TyG index for the GERD group was significantly higher than that for the control group (9.6 vs. 9.3, *P* < 0.001). The proportions of Q3 and Q4 groups in the TyG index were significantly higher in the GERD group than in the control group (30.4% vs. 25.1% in the Q3 group, and 37.4% vs. 20.4% in the Q4 group, *P* < 0.001).

**Table 2:** Univariate analysis of the risk factors for GERD.

Characteristics	Control (n = 49,532)	GERD (n = 3073)	*P* value
Age, median (IQR), years	50.0 (42.0–57.0)	49.0 (41.0–57.0)	0.401
Male (n, %)	25,551 (51.6)	2251 (73.3)	< 0.001
Height, median (IQR), cm	166.0 (159.5–172.6)	170.1 (163.9–175.4)	< 0.001
Weight, median (IQR), kg	64.9 (55.8–74.0)	72.5 (63.6–80.9)	< 0.001
BMI, median (IQR), kg/m^2^	23.5 (21.4–25.7)	24.9 (22.9–27.2)	< 0.001
Waist, median (IQR), cm	81.0 (73.0–88.0)	87.0 (80.0–93.0)	< 0.001
SBP, median (IQR), mmHg	120.0 (110.0–130.0)	121.0 (112.0–131.0)	< 0.001
DBP, median (IQR), mmHg	71.0 (65.0–79.0)	71.0 (65.0–78.0)	0.273
Obesity (n, %)	15,270 (30.8)	1451 (47.2)	< 0.001
TyG index, median (IQR)	9.3 (8.9–9.7)	9.6 (9.2–10.1)	< 0.001
**TyG index in quartile**			< 0.001
Q1 (n, %)	13,817 (27.9)	262 (8.5)	
Q2 (n, %)	13,164 (26.6)	726 (23.6)	
Q3 (n, %)	12,445 (25.1)	935 (30.4)	
Q4 (n, %)	10,106 (20.4)	1150 (37.4)	
Current smoker (n, %)	17,253 (34.8)	1629 (53.0)	< 0.001
Alcohol history (n, %)	28,780 (58.1)	2246 (73.1)	< 0.001
Hypertension (n, %)	5,173 (10.4)	594 (19.3)	< 0.001
*H. pylori* infection (n, %)	13,610 (27.5)	654 (21.3)	< 0.001
**Laboratory finding, median (IQR)**			
Fasting glucose, mg/dL	97.0 (91.0–105.0)	99.0 (93.0–108.0)	< 0.001
Total cholesterol, mg/dL	201.0 (177.0–227.0)	202.0 (177.0–228.0)	0.006
Triglyceride, mg/dL	108.0 (78.0–157.0)	125.0 (87.0–181.0)	< 0.001
HDL cholesterol, mg/dL	55.0 (47.0–64.0)	53.0 (45.0–62.0)	< 0.001
LDL cholesterol, mg/dL	131.0 (110.0–153.0)	123.0 (103.0–145.0)	< 0.001

*IQR* interquartile range; *BMI* body mass index; *SBP* systolic blood pressure; *DBP* diastolic blood pressure; *TyG index* triglyceride-glucose index; *Q* quartile; *H. pylori Helicobacter pylori*; *HDL* high-density lipoprotein; *LDL* low-density lipoprotein; *GERD* gastroesophageal reflux disease.

In multivariable analysis, the TyG index (*P* < 0.001), male sex (odds ratio [OR] = 1.425, 95% confidence interval [CI]: 1.285–1.577, *P* < 0.001), obesity (OR = 1.300, 95% CI: 1.201–1.406, *P* < 0.001), smoking (OR = 1.136, 95% CI: 1.036–1.245, *P* = 0.007), alcohol consumption (OR = 1.488, 95% CI: 1.359–1.629, *P* < 0.001), and hypertension (OR = 1.546, 95% CI: 1.403–1.703, *P* < 0.001) were identified as independent risk factors for GERD (Table 3). *H. pylori* infection was significantly negatively associated with GERD (OR = 0.678, 95% CI: 0.620–0.742, *P* < 0.001). Taking Q1 as the reference, the ORs for GERD were increased according to the TyG levels for Q2, Q3, and Q4: Q2 (OR = 2.477, 95% CI: 2.142–2.863, *P* < 0.001), Q3 (OR = 3.013, 95% CI: 2.610–3.478, *P* < 0.001), and Q4 (OR = 4.027, 95% CI: 3.484–4.655, *P* < 0.001).

**Table 3:** Multivariate analysis of the risk factors for GERD.

	OR (95% CI)	*P* value
Male	1.425 (1.285–1.577)	< 0.001
Obesity	1.300 (1.201–1.406)	< 0.001
Current smoker	1.136 (1.036–1.245)	0.007
Alcohol history	1.488 (1.359–1.629)	< 0.001
Hypertension	1.546 (1.403–1.703)	< 0.001
*H. pylori* infection	0.678 (0.620–0.742)	< 0.001
**TyG index in quartile**		< 0.001
Q1	1	
Q2	2.477 (2.142–2.863)	< 0.001
Q3	3.013 (2.610–3.478)	< 0.001
Q4	4.027 (3.484–4.655)	< 0.001

*H. pylori Helicobacter pylori*; *TyG index* triglyceride-glucose index; *Q* quartile; *OR* odds ratio; *CI* confidence interval.

### Risk factors for ERD

In the univariate analysis, male sex, obesity, smoking, alcohol consumption, and hypertension were significantly associated with an increased risk for ERD (Table 4). The median TyG index for the ERD group was significantly higher than that for the control group (9.8 vs. 9.3, *P* < 0.001). The TyG indices for the Q3 and Q4 groups were considerably higher in the ERD group than in the control group (29.0% vs. 25.1% in Q3 and 47.5% vs. 20.4% in Q4, *P* < 0.001).

**Table 4:** Univariate analysis of the risk factors for ERD.

Characteristics	Control (n = 49,532)	ERD (n = 434)	*P* value
Age, median (IQR), years	50.0 (42.0–57.0)	51.0 (43.0–58.0)	0.302
Male (n, %)	25,551 (51.6)	393 (90.6)	< 0.001
Height, median (IQR), cm	166.0 (159.5–172.6)	171.3 (166.7–176.1)	< 0.001
Weight, median (IQR), kg	64.9 (55.8–74.0)	76.7 (69.1–85.3)	< 0.001
BMI, median (IQR), kg/m^2^	23.5 (21.4–25.7)	25.9 (24.2–28.4)	< 0.001
Waist, median (IQR), cm	81.0 (73.0–88.0)	90.0 (85.0–96.0)	< 0.001
SBP, median (IQR), mmHg	120.0 (110.0–130.0)	123.5 (115.0–133.0)	< 0.001
DBP, median (IQR), mmHg	71.0 (65.0–79.0)	73.0 (67.0–79.0)	< 0.001
Obesity (n, %)	15,270 (30.8)	274 (63.1)	< 0.001
TyG index, median (IQR)	9.3 (8.9–9.7)	9.8 (9.4–10.2)	< 0.001
**TyG index in quartile**			< 0.001
Q1 (n, %)	13,817 (27.9)	14 (3.2)	
Q2 (n, %)	13,164 (26.6)	88 (20.3)	
Q3 (n, %)	12,445 (25.1)	126 (29.0)	
Q4 (n, %)	10,106 (20.4)	206 (47.5)	
Current smoker (n, %)	17,253 (34.8)	294 (67.7)	< 0.001
Alcohol history (n, %)	28,780 (58.1)	334 (77.0)	< 0.001
Hypertension (n, %)	5173 (10.4)	104 (24.0)	< 0.001
*H. pylori* infection (n, %)	13,610 (27.5)	124 (28.6)	0.611
**Laboratory finding, median (IQR)**			
Fasting glucose, mg/dL	97.0 (91.0–105.0)	102.0 (95.0–111.0)	< 0.001
Total cholesterol, mg/dL	201.0 (177.0–227.0)	206.0 (181.0–230.0)	< 0.001
Triglyceride, mg/dL	108.0 (78.0–157.0)	146.0 (100.0–207.0)	< 0.001
HDL cholesterol, mg/dL	55.0 (47.0–64.0)	50.0 (42.0–59.0)	< 0.001
LDL cholesterol, mg/dL	131.0 (110.0–153.0)	128.0 (108.0–149.8)	0.188

*IQR* interquartile range; *BMI* body mass index; *SBP* systolic blood pressure; *DBP* diastolic blood pressure; *TyG index* triglyceride-glucose index; *Q* quartile; *H. pylori Helicobacter pylori*; *HDL* high-density lipoprotein; *LDL* low-density lipoprotein; *ERD* erosive reflux disease.

In multivariable analysis, the TyG index (*P* < 0.001), male sex (OR = 3.906, 95% CI: 2.732–5.587, *P* < 0.001), obesity (OR = 1.972, 95% CI: 1.606–2.420, *P* < 0.001), smoking (OR = 1.540, 95% CI: 1.233–1.924, *P* < 0.001), and hypertension (OR = 1.747, 95% CI: 1.392–2.191, *P* < 0.001) were independent risk factors for ERD (Table 5). With respect to the TyG index, the ORs for ERD were progressively increased according to quartiles: Q2 (OR = 4.264, 95% CI: 2.418–7.522, *P* < 0.001), Q3 (OR = 4.841, 95% CI: 2.767–8.471, *P* < 0.001), and Q4 (OR = 7.390, 95% CI: 4.247–12.857, *P* < 0.001).

**Table 5:** Multivariate analysis of the risk factors for ERD.

	OR (95% CI)	*P* value
Male sex	3.906 (2.732–5.587)	< 0.001
Obesity	1.972 (1.606–2.420)	< 0.001
Current smoker	1.540 (1.233–1.924)	< 0.001
Alcohol history	1.203 (0.940–1.540)	0.142
Hypertension	1.747 (1.392–2.191)	< 0.001
**TyG index in quartile**		< 0.001
Q1	1	
Q2	4.264 (2.418–7.522)	< 0.001
Q3	4.841 (2.767–8.471)	< 0.001
Q4	7.390 (4.247–12.857)	< 0.001

*TyG index* triglyceride-glucose index; *Q* quartile; *OR* odds ratio; *CI* confidence interval.

### Progression of the TyG index and GERD

**Figure 1:**
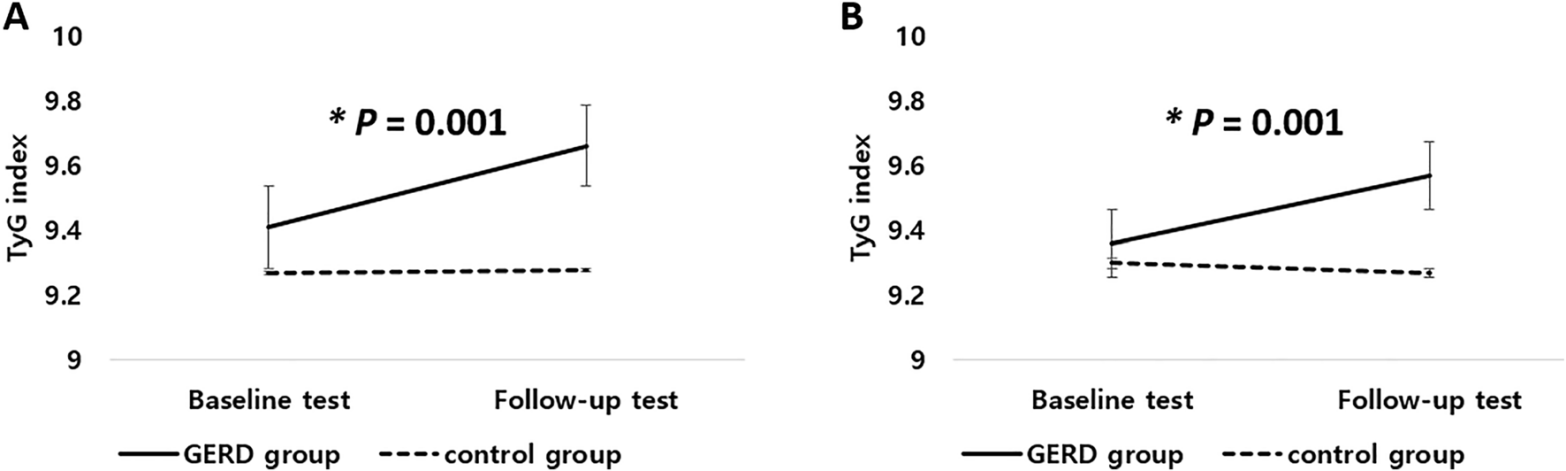
Progression of the TyG index and GERD. (**A**) Unadjusted. (**B**) Adjusted for obesity, hypertension, *H. pylori* infection, alcohol consumption, and smoking.

Among the study population, 1123 patients in the GERD group and 8815 patients in the control group received health checkups two times. The median TyG index for the GERD group was 9.3 in the first and 9.7 in the second. Moreover, the median TyG index for the control group was the same as 9.3 in the first and the second. The TyG index increase in the GERD group was more significant than in the control group (*P* = 0.001, Fig. 1A). This tendency was maintained after adjusting for obesity, hypertension, *H. pylori* infection, alcohol consumption, and smoking. After adjustment, the TyG index for the GERD group was 9.3 in the first and 9.6 in the second. The median TyG index for the control group was the same as 9.3 in the first and the second. The TyG index increase was higher in the GERD group than in the control group (*P* = 0.001, Fig. 1B).

## Discussion

To the best of our knowledge, this is the first study to demonstrate an association between the GERD, ERD, and TyG index. To evaluate IR, various methods, such as the hyperinsulinemic-euglycemic clamp and the insulin tolerance test, have been used^18,19^. Since these methods are expensive and invasive, the homeostasis model assessment for IR (HOMA-IR) was introduced as an alternative method^20^. Recently, the TyG index, a simple and cost-effective marker from routine laboratory results, was identified as a useful marker for IR in recent studies^13,21^. Despite its simplicity, the TyG index has substantial clinical importance owing to its metabolic significance.

Although the precise mechanism underlying the relationship between the TyG index and GERD is unclear, it may be explained by the metabolic syndrome, which is a complex of various metabolic conditions such as IR, obesity, dyslipidemia, and hypertension^22^. Among these conditions, IR, which is the inability of insulin to transport glucose into optimal cells, is closely associated with metabolic syndrome^23,24^.

The relationship between GERD and IR is explained by changes in esophageal motility resulting from hyperglycemia, which increases the peristaltic wave duration and decreases peristaltic velocity in the distal esophagus, decreases lower esophageal sphincter (LES) pressure, and eventually delays gastric emptying^25–27^. Pro-inflammatory cytokines have also been suggested to explain the relationship between GERD and IR. They involved disruptions in insulin transport and insulin sensitivity via cellular pathways linked with insulin receptor substrate serine phosphorylation-1 and nuclear factor-kappa B in adipocytes, hepatocytes, and macrophages^28^. Previous studies have reported that hyperglycemia is associated with a prolonged LES relaxation period compared with euglycemic conditions^25,27^, and high IR is associated with increased severity and prevalence of GERD^29^.

Several recent studies reported that serum TG levels are associated with GERD mediated via several mechanisms^30^. Intraduodenal administration of long-chain TG after meals influences the contraction and relaxations of the lower esophageal sphincter^31^, and cholesterol of dietary nutrients enhances perception of the esophagus following intraesophageal acid reflux^32^.

In addition to the glucose and TG, metabolic factors such as obesity and hypertension were significantly associated with GERD and ERD both in this study. Inflammation is involved in the association between obesity and IR. Obesity-associated inflammation in the adipose tissue and liver induces an increase in macrophage infiltration and the expression of pro-inflammatory cytokines such as tumor necrosis factor-alpha and IL-6. These cytokines, and other inflammatory mediators are involved in the insulin signaling pathway and the induction of IR. IR, with compensatory hyperinsulinemia, is an important proposed mechanism in the pathophysiology of hypertension. IR induces increased blood pressure through various mechanisms, including stimulating sympathetic nervous system activity and renal tubular sodium reabsorption. Thus, various metabolic factors and GERD are interlinked by IR.

The strength of this study was that we analyzed patients who received laboratory tests two times during the study period. The median TyG indices for the first and second tests increased in both the GERD and control groups. This result may be associated with the aging process between the first and second tests. However, the increase was more obvious in the GERD group than in the control group. This tendency was maintained after adjusting for metabolic factors, *H. pylori* infection, smoking, and alcohol consumption. An upper endoscopy may be recommended if a patient has GERD-related symptoms and shows a noticeable increase in the TyG index compared with the previous test.

GERD diagnoses are based on heterogeneous clinical symptoms such as regurgitation, heartburn, and dyspepsia^33^. Since our checkup center questionnaire did not have questions to evaluate these symptoms, GERD was diagnosed via endoscopic findings based on the LA classification^34^. To overcome this limitation, we set the “ERD group (LA grades B to D reflux esophagitis),” which is a more conclusive endoscopic finding for GERD^35^, and performed additional analysis. In addition to the TyG index, metabolic components such as obesity and hypertension were significantly associated with GERD. The TyG index and these metabolic factors were also independent predictive factors for ERD. Notably, regarding the other metabolic components and TyG index, the OR was more potent in the patients with ERD, which has a more definitive mucosal injury and is more specific than GERD. This can be also explained with the other study on GERD patients in Moscow, which found that proinflammatory cytokines levels such as IL-8 and Transforming growth factor-α (TNF-α) in serum patients with ERD were higher than those without esophagitis or normal subjects^36^.

We are also aware of several unresolved issues that should be addressed. First, because of the retrospective nature of this study, we might have collected some incomplete data. Although we handled it using a complete case analysis that excluded all patients with missing data (n = 41), it could have led to biased estimates and reduced statistical power. Second, due to the study’s cross-sectional design, there was a selection bias regarding individuals’ socioeconomic status, dietary habits, and physical activity. Furthermore, as we could not assess these data, confounding variables still needed to be considered to determine the relationship between GERD and TyG index. Third, since the enrolled patients of this study were Korean from a single center, it is hard to generalize our findings. Prospective, well-designed, longitudinal studies are required to elucidate the precise correlations between GERD, ERD, and the TyG index. Forth, GERD and ERD were diagnosed based on endoscopic findings via a retrospective review of endoscopic images in our electronic medical records. To overcome this limitation, we performed an additional analysis in the ERD group, which is a more specific mucosal injury for GERD. Finally, since the laboratory tests performed at our health checkup center did not include a test for the insulin level, the HOMA-IR was not considered. Further studies are needed to measure insulin levels and to compare the TyG index with the HOMA-IR.

In conclusion, the TyG index may be a novel predictive biomarker for GERD. Notably, the amount of the increase in the TyG index between the first and second tests was prominent for GERD. Therefore, the TyG index from routine laboratory tests could help diagnose GERD in clinical practice. Moreover, treatment of high TG or glucose levels, which are determinants of the TyG index, may be important for preventing and treating GERD.

## Methods

### Study design and population

**Figure 2:**
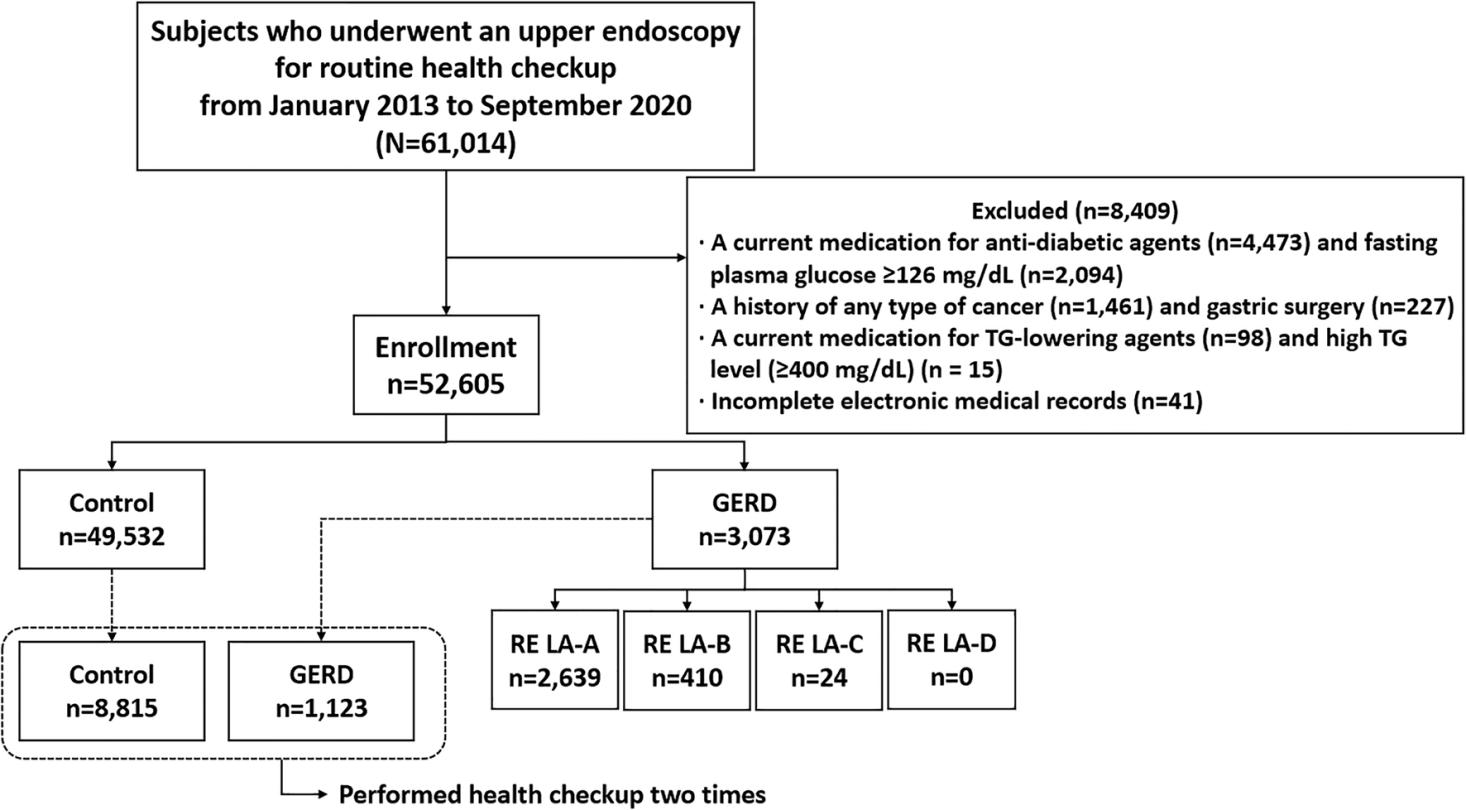
Flow chart of the enrolled patients.

We reviewed the electronic medical records of 61,014 adult patients (age > 18 years) who underwent upper gastrointestinal endoscopy for a routine health checkup from January 2013 to September 2020. Patients with a current medication for anti-diabetic agents (n = 4473) and fasting plasma glucose ≥ 126 mg/dL (n = 2094), a history of cancer (n = 1461) and gastric surgery (n = 227), a current medication for TG-lowering agents (n = 98) and high TG level (≥ 400 mg/dL) (n = 15), and incomplete electronic medical records (n = 41) were excluded. As a result, 52,605 patients were enrolled in this study (Fig. 2).

The study protocol conformed to the ethical guidelines of the World Medical Association Declaration of Helsinki and was approved by the Institutional Review Board of Gangnam Severance Hospital (IRB no. 3–2020-0395). Moreover, the informed consent was waived by the Institutional Review Board of Gangnam Severance Hospital, as this study was a retrospective analysis of the existing administrative and clinical data.

### Data collection

Data were retrospectively collected from the electronic medical records of the enrolled patients. Patients underwent a health checkup after fasting for 12 h, and the data included laboratory and anthropometric parameters and endoscopic findings. Laboratory data included fasting glucose, total cholesterol, TG, high-density lipoprotein (HDL) cholesterol, and low-density lipoprotein cholesterol levels. After overnight fasting for more than 12 h, blood samples collected from each participant were processed and transported in cold storage to the testing department. All blood samples were analyzed within 24 h after transportation. The calculation of TyG index used following formula: ln (fasting triglycerides [mg/dL] × fasting glucose [mg/dL]/2). According to the TyG index, enrolled patients were classified into four groups as follows: quartile 1 (Q1), between 6.45 and 8.93; Q2, between 8.93 and 9.30; Q3, between 9.30 and 9.72; and Q4, between 9.72 and 13.52. Moreover, to evaluate the relationship between the variation of the TyG index and GERD, we compared the TyG index between the first and second tests in patients who received health checkups twice during the study period. In the case of GERD, a follow-up test was defined as a test occurring at the time of GERD diagnosis.

Height, body weight, and waist circumference were measured while the patients wore lightweight clothing without shoes. Body mass index (BMI) was calculated as body weight divided by height squared (kg/m^2^). Obesity was defined as a BMI ≥ 25 kg/m^2^ according to the Asia–Pacific criteria^37^.

### Endoscopic evaluation of the stomach

All enrolled patients underwent upper endoscopic examinations using an endoscope (GIF-H260; Olympus Medical Systems, Tokyo, Japan) equipped with an electronic endoscopy system (EVIS LUCERA; Olympus Medical Systems). In this study, GERD was diagnosed based on endoscopic findings, including Los Angeles (LA) grades A to D reflux esophagitis. LA grades B to D were defined as erosive reflux disease (ERD).

The following three methods were used for *H. pylori* infection: rapid urease test (CLO test; Delta West, Bentley, Australia), pathology (Giemsa staining), and immunoglobulin G test specific for *H. pylori* in serum (enzyme-linked fluorescence assay, Vidas (bioMerieux Vitek, Inc. (Hazelwood, MO, USA). *H. pylori* infection was diagnosed when the results of at least one of these three tests were positive.

### Questionnaire

All patients who visited our checkup center were asked to complete the questionnaire (Supplemnatry Information 3). The questionnaire included questions on smoking, alcohol consumption, and medical history, such as hypertension. A current smoker was defined as a patient who is currently smoking or has smoked 100 cigarettes in their lifetime. Alcohol consumption was defined as the consumption of alcohol at least twice per week for a year. Patients were asked to check “yes” or “no” to indicate whether they were taking medication for hypertension. A patient was considered to have hypertension if they answered “yes” on the questionnaire when asked whether they have hypertension or if they had a systolic blood pressure ≥ 140 mmHg or a diastolic blood pressure ≥ 90 mmHg^38^.

### Statistical analysis

Patient characteristics were described as median (IQR) for continuous variables, and percentages for categorical variables. Student’s t-test or Mann–Whitney-U test was used to compare quantitative variables. The chi-squared test or Fisher’s exact test was used to compare qualitative variables. The Kaplan–Meier method was used to determine whether the TyG index might be used to predict GERD (Supplementary Infomation 1, 2). Multivariable logistic regression analyses were performed to determine the independent association between GERD/ERD and the TyG index. The confounding variables (all categorical variables), including obesity, hypertension, *H. pylori* infection, alcohol consumption, and smoking, were adjusted for comparing the TyG index increase between the GERD and the control group. Due to a small number of missing data, we used a complete case analysis that excluded all patients with any missing data (n = 41). A *P* < 0.05 was considered indicative of statistical significance. Statistical analyses were performed using Statistical Package for the Social Sciences version 25.0 for Windows (IBM Corp., Armonk, NY, USA).

## Supplementary Information

Supplementary Information 1.

Supplementary Information 2.

Supplementary Information 3.

## Data Availability

The datasets used and analyzed during the current study are available from the corresponding author on reasonable request since the datasets include patients’ personal information.
