# Supplementary figures and images for: Triglyceride-glucose index is associated with gastroesophageal reflux disease and erosive reflux disease: a health checkup cohort study
Source: Sci Rep. 2022 Dec 5;12:20959. doi: 10.1038/s41598-022-25536-0 (PMC9722682; doi:10.1038/s41598-022-25536-0)

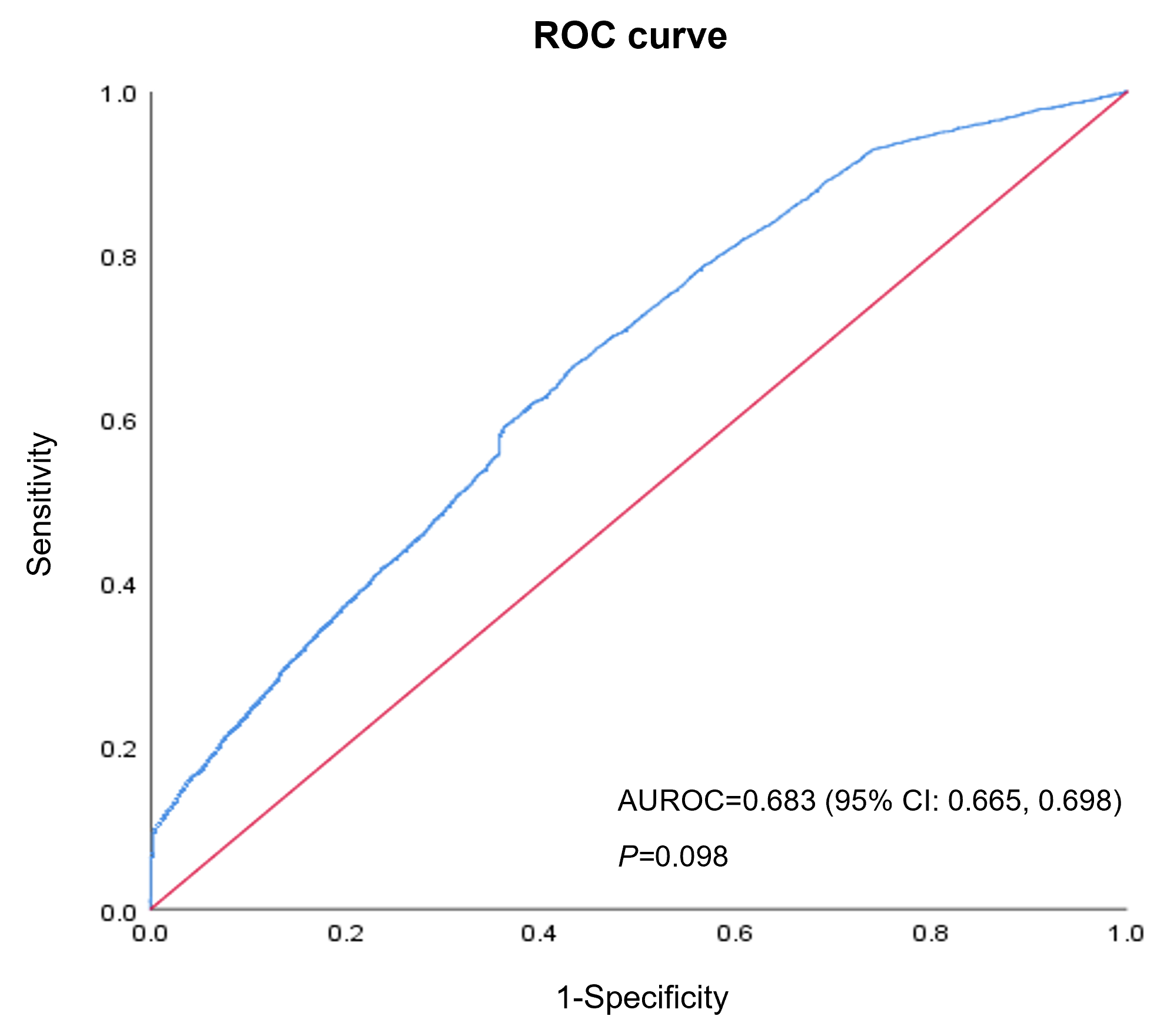

Supplement: Supplementary file 1 — Supplementary Information 1. [file 41598_2022_25536_MOESM1_ESM.tif]
