# Supplementary material for: Triglyceride-glucose index is associated with gastroesophageal reflux disease and erosive reflux disease: a health checkup cohort study
Source: Sci Rep. 2022 Dec 5;12:20959. doi: 10.1038/s41598-022-25536-0 (PMC9722682; doi:10.1038/s41598-022-25536-0)
Supplement: Supplementary file 2 — Supplementary Information 2. [file 41598_2022_25536_MOESM2_ESM.docx]

**Figure legend**

Supplementary figure 1. A ROC curve for predicting GERD using the triglyceride-glucose index. GERD, gastroesophageal reflux disease; ROC, receiver operating characteristic.
