# Supplementary material for: Triglyceride-glucose index is associated with gastroesophageal reflux disease and erosive reflux disease: a health checkup cohort study
Source: Sci Rep. 2022 Dec 5;12:20959. doi: 10.1038/s41598-022-25536-0 (PMC9722682; doi:10.1038/s41598-022-25536-0)
Supplement: Supplementary file 3 — Supplementary Information 3. [file 41598_2022_25536_MOESM3_ESM.pdf]

+1001+ +1001+

| 바코드 | |
|---|---|

강남세브란스헬스체크업

HEALTH-CHECKUP GUIDE

# 건강문진표

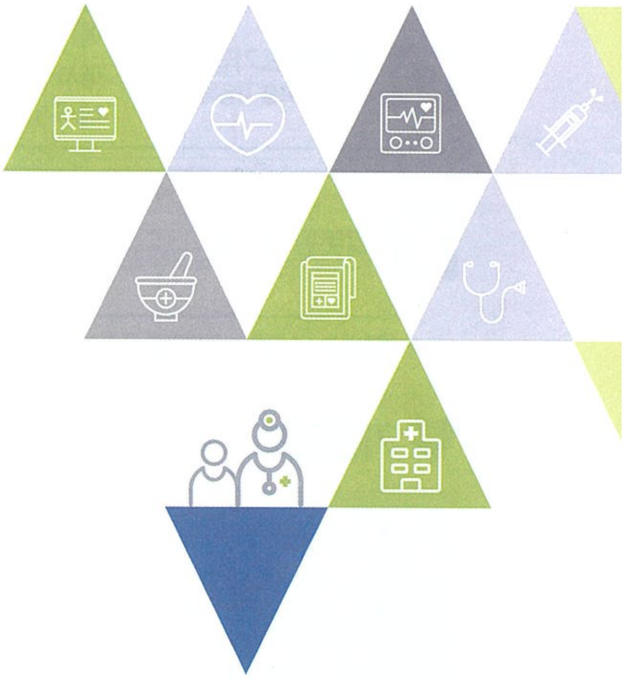

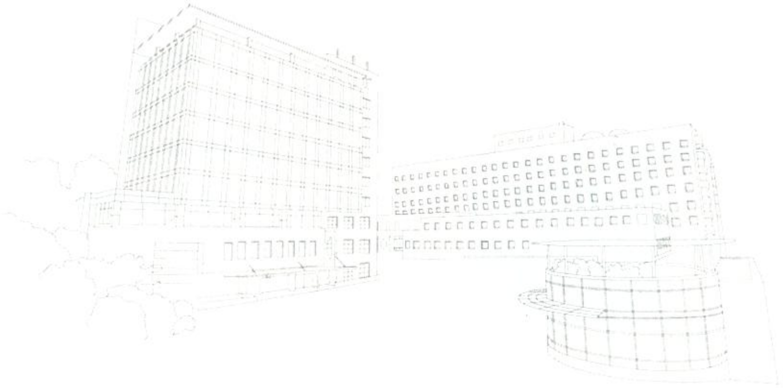

| 성 명 | |
|---|---|

※ 문진표는 OMR로 처리되므로, 구기거나 찢지 마십시오.
※ 스캔의 편의성을 위해 절취선이 들어가 있는 서식 입니다. 작성 중 페이지가 분리 될수 있습니다.
※ 문진표가 분리될 경우 스테플러, 테이프, 풀을 이용하지 말고, 클립을 이용하여 고정 후 지참해 주십시오.
※ 임신중에는 검진을 받을 수 없습니다. 임신이 의심될 경우 검사전 알려주시기 바랍니다.
※ 결과지 판정소요기간은 약 7일이며, 검사항목별 특성에 따라 다소 지연될 수도 있습니다.

**검진 시 미리 작성 하시어 꼭 지참해 주세요!!**

Gangnam Severance Health Check-up
강남세브란스헬스체크업

## 문진표 작성 요령

- 검정색 볼펜이나 싸인펜을 사용해 주십시오. ⇒ 반드시 정자로 표시해 주십시오.
- [ ][ ] : 빈 칸에는 숫자를 기입하시면 됩니다. 예) 몇 년간 [illegible]우셨습니까? [ ][3] 년 (십단위 일단위)
- 이 문진표는 헬스체크업 전문의가 귀하의 건강상태를 정확히 파악하여 검진 결과를 평가하는데 이용하는 중요한 자료이므로 빠짐없이 기록해 주시기 바랍니다.

**표기는 검정색볼펜으로 네모칸 □안에 진하고 정확하게 표시해 주십시오.**

**O 올바른 표기법** (수정테이프를 사용해 주세요.)

예) 하루 세 끼 식사를 하십니까?

예시 1) ■1년 이내 예시 2) ◉1년 이내
예시 3) ☒1년 이내 예시 4) ⍁1년 이내

**X 잘못된 표기법**

예시 1) ☑1년 이내 — 체크박스 안으로 표시해 주십시오.
예시 2) — 아래 체크칸에 닿지 않게 해 주십시오.
예시 3) — 옆의 체크칸에 닿지 않게 해 주십시오.
예시 4) — 잘못 표기시 꼭! 수정테이프를 사용해 주십시오.

※ 문진표는 OMR로 처리되므로 구기거나 찢지 마십시오.

## 기본정보

| | | | |
|---|---|---|---|
| 성 명 | | 생년월일 | □남 □여 |
| 연 령 | 만 세 | 핸 드 폰 | |
| 주 소 | | | |
| 검 진 일 | 20 년도 월 일 요일 | 이 메 일 | @ |
| 현 재 결혼 상태 | □미혼 □기혼 □별거/이혼/사별<br>□기타 □무응답 | | |
| 직 종 | □생산직 □서비스직 □영업판매직<br>□사무관리직 □전문직 □학생<br>□전업주부 □무직 □기타( ) | | |
| 최종학력 | □고졸 이하 □대학교 재학 및 중퇴 □대졸<br>□대학원 이상 □무응답 | | |
| 월 소 득 | □300만원 이하 □300~500만원 □500~700만원<br>□700~1000만원 □1000만원 이상 □무응답 | | |

+1003+

표기는 검정색볼펜으로 네모칸 □안에 진하고 정확하게 표시해 주십시오.

올바른 표기법
※수정테이프를 이용해 수정 가능

예) 하루 세 끼 식사를 하십니까?
예시 1) ■ 예시 2) ◙ 예시 3) ☒ 예시 4) ◩

+1003+

## 현병력 및 과거력 (반드시 정자로 표기해 주십시오.)

(1) 다음 아래 암중 현재 발병했거나 과거 발병했던 적이 있습니까?

□ 없다 □ 있다 (※ 있다면 아래 '해당 암'에만 ① ~ ③까지 모두 표기해 주세요)

| ① 발병 했던 암 | ② 완치 판정 여부 | ③ 발병년도 |
|---|---|---|
| □ 위암 | □ 예 □ 아니오 | □□□□ 년 |
| □ 직장암/대장암 | □ 예 □ 아니오 | □□□□ 년 |
| □ 식도암 | □ 예 □ 아니오 | □□□□ 년 |
| □ 간암 | □ 예 □ 아니오 | □□□□ 년 |
| □ 췌장암 | □ 예 □ 아니오 | □□□□ 년 |
| □ 담도암/담낭암 | □ 예 □ 아니오 | □□□□ 년 |
| □ 폐암 | □ 예 □ 아니오 | □□□□ 년 |
| □ 유방암 | □ 예 □ 아니오 | □□□□ 년 |
| □ 자궁경부암 (여성만 해당) | □ 예 □ 아니오 | □□□□ 년 |
| □ 난소암 (여성만 해당) | □ 예 □ 아니오 | □□□□ 년 |
| □ 전립선암 (남성만 해당) | □ 예 □ 아니오 | □□□□ 년 |
| □ 갑상선암 | □ 예 □ 아니오 | □□□□ 년 |
| □ 기타 암 ( ) | □ 예 □ 아니오 | □□□□ 년 |
| | | 천단위 백단위 십단위 일단위 |

(2) 다음 아래 질환중 현재 발병했거나 과거 발병했던 적이 있습니까? (다음 페이지에 계속)

□ 없다 □ 있다 (※ 있다면 아래 '해당 질환'에만 ① ~ ③까지 모두 표기해 주세요)

| ① 진단 받은 질환 | ② 현재 치료 받고 있습니까? | ③ 발병년도 |
|---|---|---|
| □ 고혈압 | □ 예 □ 아니오 | □□□□ 년 |
| □ 당뇨 | □ 예 □ 아니오 | □□□□ 년 |
| | | 천단위 백단위 십단위 일단위 |

+1004+ +1004+

| ① 진단 받은 질환 | ② 현재 치료 받고 있습니까? | ③ 발병년도 |
|---|---|---|
| ☐ 고지혈증 | ☐ 예 ☐ 아니오 | 년 |
| ☐ 심장질환 (심근경색/협심증) | ☐ 예 ☐ 아니오 | 년 |
| ☐ 심장질환 (부정맥) | ☐ 예 ☐ 아니오 | 년 |
| ☐ 심장질환 (기타) | ☐ 예 ☐ 아니오 | 년 |
| ☐ 뇌혈관질환 (뇌경색) | ☐ 예 ☐ 아니오 | 년 |
| ☐ 뇌혈관질환 (뇌출혈) | ☐ 예 ☐ 아니오 | 년 |
| ☐ 뇌혈관질환 (뇌동맥류) | ☐ 예 ☐ 아니오 | 년 |
| ☐ 갑상선 기능항진증 | ☐ 예 ☐ 아니오 | 년 |
| ☐ 갑상선 기능저하증 | ☐ 예 ☐ 아니오 | 년 |
| ☐ 갑상선 결절 | ☐ 예 ☐ 아니오 | 년 |
| ☐ 폐질환 (결핵) | ☐ 예 ☐ 아니오 | 년 |
| ☐ 폐질환 (천식) | ☐ 예 ☐ 아니오 | 년 |
| ☐ 간질환 (B형간염) | ☐ 예 ☐ 아니오 | 년 |
| ☐ 간질환 (C형간염) | ☐ 예 ☐ 아니오 | 년 |
| ☐ 간질환 (간경변) | ☐ 예 ☐ 아니오 | 년 |
| ☐ 전립선비대증 | ☐ 예 ☐ 아니오 | 년 |
| ☐ 우울증 | ☐ 예 ☐ 아니오 | 년 |
| ☐ 정신과 질환 | ☐ 예 ☐ 아니오 | 년 |
| ☐ 기타 질환 ( ) | ☐ 예 ☐ 아니오 | 년 |
| | | 천단위 백단위 십단위 일단위 |

표기는 검정색볼펜으로 네모칸 □안에 진하고 정확하게 표시해 주십시오.

올바른 표기법 ※수정테이프를 이용해 수정 가능 예) 하루 세 끼 식사를 하십니까? 예시 1) ■ 예시 2) ◙ 예시 3) ☒ 예시 4) ◪

+1005+

(3) 다음 아래 위, 대장 질환중 현재 발병했거나 과거 발병했던 적이 있습니까?

□ 없다 □ 있다 (※ 있다면 아래 '해당 질환'에만 ① ~ ③까지 모두 표기해 주세요)

| ① 진단 받은 질환 | ② 현재 치료 받고 있습니까? | ③ 진단시기 |
|---|---|---|
| □ 위, 십이지장 궤양 | □ 예 □ 아니오 | □□□□ 년 |
| □ 만성위축성 위염 | □ 예 □ 아니오 | □□□□ 년 |
| □ 장상피화생(위) | □ 예 □ 아니오 | □□□□ 년 |
| □ 헬리코박터 위염 | □ 예 □ 아니오 | □□□□ 년 |
| □ 대장용종 | □ 예 □ 아니오 | □□□□ 년 |
| □ 궤양성대장염 | □ 예 □ 아니오 | □□□□ 년 |
| □ 크론병 | □ 예 □ 아니오 | □□□□ 년 |
| □ 기타 (　　　　　　) | □ 예 □ 아니오 | □□□□ 년 |
| | | 천단위 백단위 십단위 일단위 |

## 투약력

다음 약물 중에서 의사로부터 처방 받아, 현재 복용중인 약이 있습니까?

□ 없다 □ 있다 (※ 있다면 아래 '해당 약물'에만 모두 표기해 주세요)

| | | |
|---|---|---|
| □ 혈압약 | □ 당뇨약 혹은 인슐린 | □ 고지혈증약 |
| □ 항혈소판제 (아스피린 등) | □ 항응고제 (와파린) | □ 갑상선약 |
| □ 부정맥약 | □ 여성호르몬제 | □ 진정제/수면제 |
| □ 녹내장약 | □ 전립선약 | □ 골다공증 치료약 (칼슘, 비타민D 제외) |
| □ 변비약 | □ 소염진통제 (관절염약) | □ 우울증약 |
| □ 한약/보약 | □ 영양보조식품 | □ 기타 (　　　　　　) |

## 수술력

다음 아래 수술 중에서 수술을 받은 적이 있습니까?

□ 없다 □ 있다 (※ 있다면 아래 '해당 수술'에만 모두 표기해 주세요)

| | | |
|---|---|---|
| □ 종양 (암) 수술 | □ 심장 수술 | □ 외상 및 골절 관련 수술 |
| □ 목/허리 디스크 수술 | □ 관절 질환 수술 | □ 담낭 수술 |
| □ 맹장 수술 | □ 제왕절개 (출산) | □ 자궁절제 수술 |
| □ 난소제거 수술 | □ 전립선 수술 | □ 치질 수술 |
| □ 백내장 수술 | □ 귀/코 부위 수술 (중이염, 축농증 수술 등) | □ 기타 |

## 가족력 (일반질환/암) (반드시 정자로 표기해 주십시오.)

**조부/모, 외조부/모, 부모, 형제, 자매, 자녀 중 다음 질환을 진단 받은 적이 있습니까?**

☐ 없다 ☐ 있다 (※ 있다면 아래 '해당 질환'에만 ☐에 모두 표기해 주세요)

| 구분 | 질병 | 관계 (괄호안에 해당 가족의 진단 나이를 적어주세요.) | | | | | | | |
|---|---|---|---|---|---|---|---|---|---|
| | | 부 | 모 | 형제/자매 | 자녀 | 친가 | | 외가 | |
| | | | | | | 조부 | 조모 | 조부 | 조모 |
| 일반질환 | 고혈압 | ☐( ) | ☐( ) | ☐( ) | ☐( ) | ☐( ) | ☐( ) | ☐( ) | ☐( ) |
| | 당뇨 | ☐( ) | ☐( ) | ☐( ) | ☐( ) | ☐( ) | ☐( ) | ☐( ) | ☐( ) |
| | 고지혈증 | ☐( ) | ☐( ) | ☐( ) | ☐( ) | ☐( ) | ☐( ) | ☐( ) | ☐( ) |
| | 심근경색/협심증 | ☐( ) | ☐( ) | ☐( ) | ☐( ) | ☐( ) | ☐( ) | ☐( ) | ☐( ) |
| | 뇌혈관계질환 | ☐( ) | ☐( ) | ☐( ) | ☐( ) | ☐( ) | ☐( ) | ☐( ) | ☐( ) |
| | 기타 질환 | ☐( ) | ☐( ) | ☐( ) | ☐( ) | ☐( ) | ☐( ) | ☐( ) | ☐( ) |
| 암 | 위암 | ☐( ) | ☐( ) | ☐( ) | ☐( ) | ☐( ) | ☐( ) | ☐( ) | ☐( ) |
| | 대장암/직장암 | ☐( ) | ☐( ) | ☐( ) | ☐( ) | ☐( ) | ☐( ) | ☐( ) | ☐( ) |
| | 식도암 | ☐( ) | ☐( ) | ☐( ) | ☐( ) | ☐( ) | ☐( ) | ☐( ) | ☐( ) |
| | 간암 | ☐( ) | ☐( ) | ☐( ) | ☐( ) | ☐( ) | ☐( ) | ☐( ) | ☐( ) |
| | 췌장암 | ☐( ) | ☐( ) | ☐( ) | ☐( ) | ☐( ) | ☐( ) | ☐( ) | ☐( ) |
| | 담도암/담낭암 | ☐( ) | ☐( ) | ☐( ) | ☐( ) | ☐( ) | ☐( ) | ☐( ) | ☐( ) |
| | 폐암 | ☐( ) | ☐( ) | ☐( ) | ☐( ) | ☐( ) | ☐( ) | ☐( ) | ☐( ) |
| | 유방암 | ☐( ) | ☐( ) | ☐( ) | ☐( ) | ☐( ) | ☐( ) | ☐( ) | ☐( ) |
| | 자궁경부암 | ☐( ) | ☐( ) | ☐( ) | ☐( ) | ☐( ) | ☐( ) | ☐( ) | ☐( ) |
| | 난소암 | ☐( ) | ☐( ) | ☐( ) | ☐( ) | ☐( ) | ☐( ) | ☐( ) | ☐( ) |
| | 전립선암 | ☐( ) | ☐( ) | ☐( ) | ☐( ) | ☐( ) | ☐( ) | ☐( ) | ☐( ) |
| | 갑상선 | ☐( ) | ☐( ) | ☐( ) | ☐( ) | ☐( ) | ☐( ) | ☐( ) | ☐( ) |
| | 기타암( ) | ☐( ) | ☐( ) | ☐( ) | ☐( ) | ☐( ) | ☐( ) | ☐( ) | ☐( ) |

## 성인예방접종력

**다음의 예방 접종을 받은 적이 있습니까?**

| 예방접종 | 접종 여부 |
|---|---|
| 독감 인플루엔자 (1년 이내) | ☐ 예 ☐ 아니오 ☐ 모름 |
| 폐렴구균 | ☐ 예 ☐ 아니오 ☐ 모름 |
| 대상포진 | ☐ 예 ☐ 아니오 ☐ 모름 |
| 성인용 파상풍 (10년 이내) 혹은 성인용 파상풍/백일해 | ☐ 예 ☐ 아니오 ☐ 모름 |
| 자궁경부암 바이러스(HPV) | ☐ 예 ☐ 아니오 ☐ 모름 |
| A형 간염 예방접종 혹은 항체 확인 (50세 미만) | ☐ 예 ☐ 아니오 ☐ 모름 |

## 약물 부작용

약물에 대한 알레르기가 있으면 해당 항목에 표시해 주십시오.

**□ 없다 □ 있다 (※ 있다면 아래 '해당 약물'에만 모두 표기해 주세요)**

| | | |
|---|---|---|
| □ 페니실린계 항생제 | □ 세파계 항생제 | □ 설파제(항균제) |
| □ 아스피린 | □ 피린계 약제 | □ 리도카인 |
| □ 조영제 | □ 기타 약물 ( ) | |

※ 항균제는 항생제, 항진균제 및 항생 약품을 포괄하는 의미 입니다. 다른 약품 뒤에 붙는 설파 성분과 구분하기 위하여 항균제를 표기 하였습니다.

## 검사력

가장 마지막으로 검사를 받은 때에 표시해 주십시오.

| 검사명 | 없음 | 1년 미만 | 1~2년 미만 | 2~5년 미만 | 5~10년 미만 | 10년 이상 |
|---|---|---|---|---|---|---|
| 위내시경 | □ | □ | □ | □ | □ | □ |
| 대장내시경 | □ | □ | □ | □ | □ | □ |
| 복부 초음파 | □ | □ | □ | □ | □ | □ |
| 복부 컴퓨터 단층촬영 (CT)) | □ | □ | □ | □ | □ | □ |
| 흉부 X선 검사 | □ | □ | □ | □ | □ | □ |
| 흉부 컴퓨터 단층촬영(CT)) | □ | □ | □ | □ | □ | □ |
| 갑상선 초음파 | □ | □ | □ | □ | □ | □ |
| 유방 X선 | □ | □ | □ | □ | □ | □ |
| 유방 초음파 | □ | □ | □ | □ | □ | □ |
| 부인과 초음파 | □ | □ | □ | □ | □ | □ |
| 자궁경부암 세포검사 | □ | □ | □ | □ | □ | □ |
| 전립선 특이항원 (혈액) | □ | □ | □ | □ | □ | □ |
| 전립선 초음파 | □ | □ | □ | □ | □ | □ |
| 심장 관상동맥 CT | □ | □ | □ | □ | □ | □ |
| 뇌 MRI/MRA | □ | □ | □ | □ | □ | □ |
| PET-CT | □ | □ | □ | □ | □ | □ |

## 흡연력 (반드시 정자로 표기해 주십시오.) (다음 페이지에 계속)

□ **전혀 피운 적이 없다.** (평생 100개비 미만)

□ **현재 피운다.**

1) 지금까지 담배를 몇 년간 피우셨습니까? [ ][ ] 년 (십단위 일단위)

2) 평균 하루 흡연량은 몇 개비 입니까? [ ][ ] 개비 (십단위 일단위)

☐ 피우다 끊었다.

1) 금연 전 까지 담배를 몇 년간 피우셨습니까? ☐☐ 년 (십단위 일단위)

2) 금연 전 까지 평균 하루 흡연량은 몇 개비 입니까? ☐☐ 개비 (십단위 일단위)

3) 담배를 끊은 나이는? (금연 시작하신 나이는?) ☐☐ 세 (십단위 일단위)

## 음주력 (반드시 정자로 표기해 주십시오.)

☐ **마시지 않는다.**

☐ **마신다.** 음주잔수기준 : 술 종류에 관계없이 각각의 술잔으로 계산(단, 캔맥주 1개(355cc) = 맥주 1.6잔)

| 술 종류 | 1회 음주량 | 주당 회수 | 기간(년) |
|---|---|---|---|
| ☐ 소주 | ☐☐ 잔 | ☐ 회 | ☐☐ 년 |
| ☐ 맥주 | ☐☐ 잔 | ☐ 회 | ☐☐ 년 |
| ☐ 양주 | ☐☐ 잔 | ☐ 회 | ☐☐ 년 |
| ☐ 와인 | ☐☐ 잔 | ☐ 회 | ☐☐ 년 |
| ☐ 막걸리 | ☐☐ 잔 | ☐ 회 | ☐☐ 년 |
| ☐ 기타 (　　　　　) | ☐☐ 잔 | ☐ 회 | ☐☐ 년 |
| | 십단위 일단위 | 일단위 | 십단위 일단위 |

## 운동

☐ **하지 않는다.**

☐ **하고 있다.**

1) 최근 1주일간, 평소보다 숨이 훨씬 더 차게 만드는 격렬한 활동을, 하루 20분 이상 시행한 날은 며칠이었습니까? (달리기, 등산, 에어로빅 등)

☐ 없음 ☐ 1일 ☐ 2일 ☐ 3일 ☐ 4일 ☐ 5일 ☐ 6일 ☐ 7일

2) 최근 1주일간, 평소보다 숨이 더 차게 만드는 중간정도 활동을, 하루 30분 이상 시행한 날은 며칠이었습니까? (속보, 자전거 등)

☐ 없음 ☐ 1일 ☐ 2일 ☐ 3일 ☐ 4일 ☐ 5일 ☐ 6일 ☐ 7일

3) 최근 1주일간, 한 번에 적어도 10분 이상씩 걸은 경우를 합하여, 하루 총 30분 이상 걸은 날은 며칠이었습니까? (걷기 등)

☐ 없음 ☐ 1일 ☐ 2일 ☐ 3일 ☐ 4일 ☐ 5일 ☐ 6일 ☐ 7일

# 영양

## ● 규칙적인 식생활

| | | | |
|---|---|---|---|
| 1. 하루 세 끼 식사를 하십니까? | □ 예 | □ 가끔 | □ 아니오 |
| 2. 정해진 시간에 식사를 하십니까? | □ 예 | □ 가끔 | □ 아니오 |
| 3. 한끼 식사에 걸리는 시간이 얼마나 되십니까? | □ 15분 이하 | □ 15~30분 | □ 30분이상 |
| 4. 과식을 자주 하십니까? | □ 예 | □ 가끔 | □ 아니오 |
| 5. 외식을 자주 하십니까? | □ 예 | □ 가끔 | □ 아니오 |
| 6. 물을 충분히 섭취하십니까? (1일 권장량: 1.5L~2L) | □ 예 | □ 가끔 | □ 아니오 |
| 7. 영양에 관한 정보에 관심이 있으십니까? | □ 많음 | □ 보통 | □ 적음 |

## ● 균형잡힌 식생활

| | | | |
|---|---|---|---|
| 8. 곡류(밥, 빵, 국수, 떡, 감자, 고구마, 시리얼 등)를 하루에 얼마나 드십니까? | □ 1~2회 | □ 3회 | □ 4회 |
| 9. 단백질류(생선, 고기, 계란, 콩, 두부, 멸치 등)를 하루에 얼마나 드십니까? | □ 1~2회 | □ 3회 | □ 4회 |
| 10. 채소류(나물, 생채, 해조류, 버섯, 샐러드, 쌈 등)를 하루에 얼마나 드십니까? | □ 1~2회 | □ 3회 | □ 4회 |
| 11. 식물성기름(콩기름, 올리브유, 들기름 등)을 일주일에 얼마나 드십니까? | □ 주 0~2회 | □ 주 3~5회 | □ 주 6~7회 |
| 12. 과일류(딸기, 사과, 포도 등 또는 생과일 주스 등)를 일주일에 얼마나 드십니까? | □ 주 0~2회 | □ 주 3~5회 | □ 주 6~7회 |
| 13. 우유 및 유제품류(유류, 요구르트, 요플레, 치즈 등) 또는 두유를 일주일에 얼마나 드십니까? | □ 주 0~2회 | □ 주 3~5회 | □ 주 6~7회 |

## ● 건강증진 섭취습관

| | | | |
|---|---|---|---|
| 14. 단 음식(과자, 사탕, 초콜렛, 꿀, 가공음료 등)을 일주일에 얼마나 드십니까? | □ 주 0~2회 | □ 주 3~5회 | □ 주 6~7회 |
| 15. 짠 음식(장아찌, 김치, 국물류, 젓갈, 자반 등)을 일주일에 얼마나 드십니까? | □ 주 0~2회 | □ 주 3~5회 | □ 주 6~7회 |
| 16. 기름진 음식(삼겹살, 갈비, 피자, 햄버거 등의 패스트푸드 등)을 일주일에 얼마나 드십니까 | □ 주 0~2회 | □ 주 3~5회 | □ 주 6~7회 |
| 17. 계란 노른자, 내장육(간, 곱창 등), 장어, 새우, 오징어 등을 일주일에 얼마나 드십니까? | □ 주 0~2회 | □ 주 3~5회 | □ 주 6~7회 |
| 18. 커피, 차(홍차,녹차 등)를 하루 3잔 이상 드시는 경우가 일주일에 얼마나 됩니까? | □ 주 0~2회 | □ 주 3~5회 | □ 주 6~7회 |
| 19. 지나치게 자극적이거나 매운음식을 일주일에 얼마나 드십니까? | □ 주 0~2회 | □ 주 3~5회 | □ 주 6~7회 |
| 20. 잦은 음주 및 과음하시는 경우가 일주일에 얼마나 됩니까? | □ 주 0~2회 | □ 주 3~5회 | □ 주 6~7회 |

## 일반 문진 (반드시 정자로 표기해 주십시오.)

☐ 1. 안색이 나쁘다. ☐ 2. 열이 난다. ☐ 3. 오한이 난다. ☐ 4. 식은땀이 흐른다.

☐ 5. 체중 감소 : [ ][ ] 개월동안 [ ][ ] kg
(십단위 일단위 / 십단위 일단위)

☐ 6. 체중 증가 : [ ][ ] 개월동안 [ ][ ] kg
(십단위 일단위 / 십단위 일단위)

☐ 7. 식욕감퇴가 있다. ☐ 8. 쉽게 피로해진다. ☐ 9. 해당 사항 없다.

## 호흡기계

☐ 1. 재채기, 콧물, 코 막힘이 있다.
☐ 2. 목에 통증이 있다.
☐ 3. 목에 이물감이 있다.
☐ 4. 목이 쉰다.
☐ 5. 기침이 자주 난다.
☐ 6. 가래가 끓는다.
☐ 7. 혈담, 각혈 등이 있다.
☐ 8. 해당사항 없다.

## 심혈관계

☐ 1. 호흡이 곤란하다.
☐ 2. 심장이 두근거린다.
☐ 3. 가슴에 통증이 있다.
☐ 4. 가슴이 답답하다.
☐ 5. 맥박이 불규칙하다.
☐ 6. 몸이 붓는다.
☐ 7. 어지럽고 현기증이 난다.
☐ 8. 해당사항 없다.

## 소화기계

☐ 1. 소화불량
☐ 2. 트림
☐ 3. 상복부 불쾌감
☐ 4. 공복시 위의 통증
☐ 5. 식후 위의 통증
☐ 6. 식사와 무관한 위의 통증
☐ 7. 구역질
☐ 8. 구토
☐ 9. 황달
☐ 10. 하복부 불쾌감
☐ 11. 하복부 통증
☐ 12. 변비
☐ 13. 설사
☐ 14. 배변시 통증
☐ 15. 혈변
☐ 16. 검은색 변
☐ 17. 해당사항 없음

## 내분비계

☐ 1. 갈증, 소변량이 증가했다.
☐ 2. 갑상선 비대가 있다.
☐ 3. 더위를 못 참는다.
☐ 4. 땀이 많이 난다.
☐ 5. 안면이 붉어진다.
☐ 6. 해당사항 없다.

## 혈액계

☐ 1. 현기증이 난다.
☐ 2. 코피가 잘 난다.
☐ 3. 잇몸에 피가 잘 난다.
☐ 4. 멍이 잘 든다.
☐ 5. 임파선 비대가 있다.
☐ 6. 해당사항 없다.

표기는 검정색볼펜으로 네모칸 □안에 진하고 정확하게 표시해 주십시오.

올바른 표기법
※수정테이프를 이용해 수정 가능

예) 하루 세 끼 식사를 하십니까?
예시 1) ■ 예시 2) ◙ 예시 3) ☒ 예시 4) ▨

+1011+

## 근골격계

- □ 1. 허리가 아프다.
- □ 2. 관절통이 있다.
- □ 3. 관절운동 장애가 있다.
- □ 4. 뒷목이 뻣뻣하고 아프다.
- □ 5. 어깨를 움직이기 힘들고 아프다.
- □ 6. 팔다리가 저리고 아프다.
- □ 7. 해당사항 없다.

## 비뇨기계

- □ 1. 소변을 자주 본다.(평상시)
- □ 2. 소변을 자주 본다.(야간)
- □ 3. 배뇨시 통증이 있다.
- □ 4. 소변에 피가 섞여 나온다.
- □ 5. 소변을 보기가 힘들다.
- □ 6. 소변을 못 참는다.
- □ 7. 소변량이 줄었다.
- □ 8. 해당사항 없다.

## 피부계

- □ 1. 발진이 있다.
- □ 2. 두드러기가 난다.
- □ 3. 부스럼, 종창 등이 있다.
- □ 4. 가려움증이 있다.
- □ 5. 해당사항 없다.

## 시각계

- □ 1. 시력장애가 있다.
- □ 2. 사물이 둘로 보인다.
- □ 3. 눈이 아프다.
- □ 4. 눈부심이 있다.
- □ 5. 눈곱이 자주 낀다.
- □ 6. 충혈이 자주 된다.
- □ 7. 해당사항 없다.

## 청각계

- □ 1. 청력이 감소한다.
- □ 2. 귀가 아프다.
- □ 3. 귀에서 분비물이 나온다.
- □ 4. 귀 울림이 있다.
- □ 5. 주위가 빙빙 돈다.
- □ 6. 해당사항 없다.

## 정신건강

- □ 1. 건망증이 심해졌다.
- □ 2. 잠자기가 어렵거나 너무 오래 잔다.
- □ 3. 이유 없이 불안하다.
- □ 4. 항상 우울하고 의욕이 없다.
- □ 5. 지나치게 술을 자주 마신다.
- □ 6. 해당사항 없다.

## 신경계

- □ 1. 두통증상이 있다.
- □ 2. 의식 상실이 있다.
- □ 3. 감정변화가 심하다.
- □ 4. 정신집중이 잘 안 된다.
- □ 5. 신경질이 자주 난다.
- □ 6. 기억력 감퇴가 있다.
- □ 7. 말을 하기가 힘들다.
- □ 8. 팔, 다리에 마비증세가 있다.
- □ 9. 팔, 다리에 감각이상이 있다.
- □ 10. 해당사항 없다.

# 여성 | 부인과 기록지 (반드시 정자로 표기해 주십시오.) » 여성만 작성해주세요.

1. 초경 연령은 언제입니까? (만 나이)

| | | | | |
|---|---|---|---|---|
| ☐ 초경안함 | ☐ 11세 | ☐ 13세 | ☐ 15세 | ☐ 17세 |
| ☐ 10세 이하 | ☐ 12세 | ☐ 14세 | ☐ 16세 | ☐ 18세 이상 |

2. 최근 마지막 생리 시작일은 언제였습니까?

☐☐☐☐ 년 ☐☐ 월 ☐☐ 일

천단위 백단위 십단위 일단위 / 십단위 일단위 / 십단위 일단위

3. 생리를 안하셨다면 언제 폐경 되셨습니까? ☐☐ 세

십단위 일단위

• 현재 호르몬약을 복용중이십니까? ☐ 예 (종류 : ) ☐ 아니오

4. 유산을 포함한 총 임신은 몇 번 하셨습니까?

☐ 0회 ☐ 1회 ☐ 2회 ☐ 3회 ☐ 4회 ☐ 5회 이상

5. 출산은 모두 몇 번 하셨습니까?

☐ 0회 ☐ 1회 ☐ 2회 ☐ 3회 ☐ 4회 ☐ 5회 이상

6. 현재 자녀는 모두 몇 분이십니까?

☐ 0명 ☐ 1명 ☐ 2명 ☐ 3명 ☐ 4명 ☐ 5명 이상

7. 유산은 모두 몇 번 하셨습니까?

☐ 0회 ☐ 1회 ☐ 2회 ☐ 3회 ☐ 4회 ☐ 5회 이상

8. 피임기구(예: 루프, 미레나 등)를 사용 중 이십니까? ☐ 예 (종류 : ) ☐ 아니오

9. 산부인과에서 입원이나 수술을 하셨다면 어떤 수술을 하셨습니까?

• 언제 : ☐☐☐☐ 년 / 어떤수술 : ______________________

천단위 백단위 십단위 일단위

• 결과 : ☐ 정상 ☐ 이상소견 ( )

10. 최근에 자궁경부암검사를 하셨다면 결과는 어떻게 나왔습니까?

• 언제 : ☐☐☐☐ 년

천단위 백단위 십단위 일단위

• 결과 : ☐ 정상 ☐ 이상소견 ( )

11. 현재 부인과적으로 불편한 점이 있으십니까?

□ **없다** □ **있다 (※ 있다면 아래 '해당 항목'에만 모두 표기해 주세요)**

□ 1. 월경이 불규칙하다.
□ 2. 월경량이 많거나 오래 지속된다.
□ 3. 생리통이 있다.
□ 4. 부정 출혈이 있다.
□ 5. 냉이 많다.
□ 6. 아래가 가렵다.
□ 7. 기침이나 재채기 시 소변이 샌다.
□ 8. 소변을 자주 보거나 참지 못한다.
□ 9. 질 쪽으로 불룩하게 나오는 것이 있다.
□ 10. 기타 (                    )

## ♀ 여성 | 유방검진 설문지 (반드시 정자로 표기해 주십시오.) » 여성만 작성해주세요.

1. 현재 유방 쪽으로 불편한 점이 있으십니까?

□ **없다** □ **있다 (※ 있다면 아래 '해당 항목'에만 모두 표기해 주세요)**

□ 1. 멍울이 만져진다.
□ 2. 분비물이 생긴다.
□ 3. 유즙이 분비된다.
□ 4. 통증이 있다.
□ 5. 종양이 있다.
□ 6. 젖유종이 있다.
□ 7. 기타 (                    )

2. 과거 유방수술이나 조직검사를 받아 본 적이 있습니까?

□ **없다** □ **있다 (※ 있다면 아래 '해당 항목'에만 모두 표기해 주세요)**

| | 검사일 | 검사결과 |
|---|---|---|
| □ 조직검사 | □□□□ 년 □□ 월 | |
| □ 맘모톰 | □□□□ 년 □□ 월 | |
| □ 수술 | □□□□ 년 □□ 월 | |
| | 천단위 백단위 십단위 일단위 / 십단위 일단위 | |

3. 유방 X선검사를 받아본 적이 있습니까?

□ **없다** □ **있다 (※ 있다면 아래 '해당 항목'에만 모두 표기해 주세요)**

• 마지막 검사일 : □□□□ 년 □□ 월
(천단위 백단위 십단위 일단위 / 십단위 일단위)

• 결과 : □ 정상 □ 이상소견 (                    )

4. 직계 가족 중 유방암이나 유방질환이 있습니까? □ **없다** □ **있다 (※ 있다면 아래 '해당 항목'에만 모두 표기해 주세요)**

• 질환 □ 유방암 □ 기타 유방질환 (          )

• 관계 □ 어머니 □ 여자형제 □ 이모 □ 딸

# 배뇨증상에 대한 질문서

**다음은 최근 한달 동안 소변을 어떻게 보고 있는 지를 묻는 것으로 해당 사항에 'V' 표기를 해주십시오.**

| | 전혀 없다 | 드물다 | 가끔 | 절반 정도 | 절반 이상 | 항상 |
|---|---|---|---|---|---|---|
| 1. 소변을 본 후 시원하지 않고 남아있는 느낌이 있습니까? | ☐0 | ☐1 | ☐2 | ☐3 | ☐4 | ☐5 |
| 2. 배뇨 후 2시간이 채 지나기 전에 또 소변을 보는 경우가 있습니까? | ☐0 | ☐1 | ☐2 | ☐3 | ☐4 | ☐5 |
| 3. 배뇨 중 오줌줄기가 끊어졌다가 힘을 주면 다시 나오는 경우가 있습니까? | ☐0 | ☐1 | ☐2 | ☐3 | ☐4 | ☐5 |
| 4. 소변이 마려울 때 참기가 어려운 경우가 얼마나 자주 있습니까? | ☐0 | ☐1 | ☐2 | ☐3 | ☐4 | ☐5 |
| 5. 배뇨 시 오줌줄기가 약하다고 느껴지는 경우는 얼마나 자주 있습니까? | ☐0 | ☐1 | ☐2 | ☐3 | ☐4 | ☐5 |
| 6. 소변이 마려운데도 바로 나오지 않고 한참 기다려야 나오는 경우가 있습니까? | ☐0 | ☐1 | ☐2 | ☐3 | ☐4 | ☐5 |
| 7. 밤에 주무시는 동안 평균 몇 번 정도 깨어서 소변을 보십니까? | ☐0 없음 | ☐1 1번 | ☐2 2번 | ☐3 3번 | ☐4 4번 | ☐5 5번 이상 |

8. 만일 지금과 같은 배뇨상태가 지속된다면 어떻게 생각되십니까?

☐ 매우 만족한다. ☐ 만족한다. ☐ 대체로 만족한다. ☐ 그저 그렇다.
☐ 대체로 불편하다. ☐ 매우 불편하다. ☐ 이 상태로는 못 참겠다.

**최근 1주일간 상태중 가장 가까운 것을 하나만 골라서 'V' 표기를 해주십시오.**

1. 아침에 일어나서 밤에 자기전까지 몇 회 정도 소변을 보셨습니까?

☐ 0(7회이하) ☐ 1(8~14회) ☐ 2(15회 이상)

2. 밤에 잠든 후부터 아침에 일어날때가지 소변을 보기위해 몇 회나 일어나셨습니까?

☐ 0(0회) ☐ 1(1회) ☐ 2(2회) ☐ 3(3회)

3. 갑자기 소변이 마려워 참기 힘들었던 적이 있었습니까?

☐ 0(없음) ☐ 1(일주일에 1회보다는 적음) ☐ 2(일주일에 1회 또는 그이상)
☐ 3(1일 1회정도) ☐ 4(1일 2~4회) ☐ 5(1일 5회 또는 그이상)

4. 갑자기 소변이 마려워서 참지 못한고 소변을 지린 적이 있었습니까?

☐ 0(없음) ☐ 1(일주일에 1회보다는 적음) ☐ 2(일주일에 1회 또는 그이상)
☐ 3(1일 1회정도) ☐ 4(1일 2~4회) ☐ 5(1일 5회 또는 그이상)

## 검진을 받게 된 동기

□ 1. 최근 건강에 자신을 갖지 못해서
□ 2. 최근 건강에 이상을 느껴서
□ 3. 정기적으로 검진
□ 4. 의사의 권유
□ 5. 회사동료의 권유
□ 6. 친척 친지의 권유
□ 7. 직장단체 검진
□ 8. 기타

## 본원 헬스체크업을 어떻게 알게 되셨습니까?

□ 1. 인터넷 검색을 통한 정보획득
□ 2. 주변 추천을 통해
□ 3. 원내 홍보물을 통해
□ 4. 병원 셔틀버스 부착 홍보물을 통해
□ 5. 기타

## 본원 헬스체크업을 선택한 이유는 무엇입니까?

□ 1. 검진프로그램이 맘에 들어서
□ 2. 연세의료원이 주는 신뢰성 때문에
□ 3. 교직원이 추천해 주셔서
□ 4. 본원에서 검진받으신 분이 추천해 주셔서
□ 5. 매년 검진을 받았던 병원이라서
□ 6. 직장에서 지정한 검진기관이어서
□ 7. 기타

## 동의서

• 건강검진 결과는 질병예방 및 건강증진을 위한 임상연구에 중요한 자료입니다.

• 연구 수행시 수진자의 사생활보호와 기밀유지를 최우선으로 하고, 개인정보 보호의 의무를 준수합니다.

• 귀하의 건강검진 결과 및 문진자료는 연구목적 이외의 다른 용도로 사용되지 않습니다.

• 귀하가 원하지 않을 경우 언제든지 연구참여를 철회할 수 있으며 이에 따른 어떠한 불이익도 받지 않습니다.

### 다음 각 항목에 대해 동의 여부를 해당란에 표시하여 주십시오.

| | | |
|---|---|---|
| 1. 본인은 동의서의 내용을 잘 이해하였으며, 자발적으로 이 연구에 참여하는 것에 동의합니다. | □ 동의함 | □ 동의하지 않음 |
| 2. 본인은 건강문진조사와 검진의 결과정보가 연구의 목적으로 이용되는 것에 동의합니다. | □ 동의함 | □ 동의하지 않음 |
| 3. 본인은 원하지 않을 경우 언제든지 연구참여를 철회할 수 있으며 이에 따른 어떠한 불이익도 받지 않는다는 것을 이해하고 있습니다. | □ 동의함 | □ 동의하지 않음 |

성 명 ____________ 서명 작성일 ________ 년 ______ 월 ______ 일

# Thank You!!

장시간 수고하셨습니다.
알려주신 소중한 자료를 기반으로 귀하의 건강을
지켜드릴 수 있는 결과와 내용을 드리도록 노력하겠습니다.

감 · 사 · 합 · 니 · 다.

Gangnam Severance Health Check-up
강남세브란스헬스체크업

서울특별시 강남구 언주로 211 강남세브란스병원 1동 4층
문의 : 1899-7588 http://gs.iseverance.com/health
